# Chrono-Endocrinology in Clinical Practice: A Journey from Pathophysiological to Therapeutic Aspects

**DOI:** 10.3390/life14050546

**Published:** 2024-04-24

**Authors:** Silvia Mercadante, Antonio Bellastella

**Affiliations:** Department of Cardiothoracic and Respiratory Sciences, University of Campania “Luigi Vanvitelli”, 80131 Naples, Italy; silvia.mercadante@studenti.unicampania.it

**Keywords:** circadian clocks, endocrine rhythms, rhythm disruption, chronotherapy

## Abstract

This review was aimed at collecting the knowledge on the pathophysiological and clinical aspects of endocrine rhythms and their implications in clinical practice, derived from the published literature and from some personal experiences on this topic. We chose to review, according to the PRISMA guidelines, the results of original and observational studies, reviews, meta-analyses and case reports published up to March 2024. Thus, after summarizing the general aspects of biological rhythms, we will describe the characteristics of several endocrine rhythms and the consequences of their disruption, paying particular attention to the implications in clinical practice. Rhythmic endocrine secretions, like other physiological rhythms, are genetically determined and regulated by a central hypothalamic CLOCK located in the suprachiasmatic nucleus, which links the timing of the rhythms to independent clocks, in a hierarchical organization for the regulation of physiology and behavior. However, some environmental factors, such as daily cycles of light/darkness, sleep/wake, and timing of food intake, may influence the rhythm characteristics. Endocrine rhythms are involved in important physiological processes and their disruption may cause several disorders and also cancer. Thus, it is very important to prevent disruptions of endocrine rhythms and to restore a previously altered rhythm by an early corrective chronotherapy.

## 1. Introduction

Rhythmic activity is a fundamental property of living matter that persists in constant environmental conditions, as it is the result of interaction between an individual genetically induced chrono-organization and the cyclical variations in some environmental factors. Recent studies have contributed to clarifying the mechanisms underlying the biological chrono-organization in animals and humans, and their results are gaining increasing relevance in Medicine [1,2]. The chrono-endocrinology studies the hormonal rhythms and their importance in physiology, pathophysiology and clinical practice. Even if mammalian circadian rhythms, including endocrine oscillations, are genetically determined, they can be influenced by environmental and epigenetic factors. Disruption of the rhythmic organization may promote the onset of important diseases including cancer [1,2,3,4]. The aim of this review is first, to summarize the general aspects of biological rhythms, drawing attention to genetic, epigenetic and environmental factors involved in their regulation; second, to describe the characteristics of several endocrine rhythms, discussing their role in physiological processes, the alterations caused by their disruption and the implications for clinical practice; third, to discuss whether such disruption may be reversed, and if possible how to plan a successful chronotherapy. To this end, we review the results of some personal experiences and of original and observational studies, reviews, meta-analyses and case reports, published up to March 2024, according to the PRISMA guidelines and the combination of these keywords: circadian, ultradian, infradian endocrine rhythms, rhythm disruption, chronophysiology, chronopathology, and chronotherapy.

## 2. General Aspects of Biological Rhythms

### 2.1. Rhythm Parameters

The parameters of a biological rhythm are the following: Period, phase, medium level or mesor, and amplitude.

*Period:* this parameter indicates the duration of a complete cycle of a rhythmic variable. If we consider, for example, the period of a hormonal rhythm, this goes from the time of the starting value to the return to the basal value after reaching the highest value (zenith or acrophase of the rhythm). Taking into account their period, rhythms are classified as circadian, if their period ranges from 24 ± 4 h, ultradian, if their period is <20 h, and infradian, if their period is >28 h. Infradian rhythms are classified as circaseptan, circatrigintan and circannual for periods of approximately 7, 30, and 360 days, respectively.

*Phase* is given by the instantaneous value of a rhythmic physiological variable at a predetermined time. It can be referred to as time detection in relation to the particular system used to express the period (for example, if the period of a rhythm is indicated in degrees, the full duration of the period will be indicated as 360°).

*Medium level or mesor* is expressed by the mean value of the rhythmic variable. If we consider, for example, a hormonal rhythm, it is expressed by the average of all hormonal values of this rhythm). 

*Amplitude* is the maximum deviation from the medium level of the rhythmic variable investigated [5,6,7,8,9].

### 2.2. Molecular Circadian Machinery

Physiological processes in animal and humans are rhythmically modulated at molecular level by a chain of circadian clocks. The main gene, the so-called “CLOCK” gene, is located at hypothalamic level in the suprachiasmatic nucleus (SCN). This gene, from this site, dictates the timing of the rhythms to central and peripheral clock genes, which contribute, in a hierarchical way, at orchestrating the physiological rhythmic activity [2,10,11,12]. The role of the main CLOCK, biochemically identified as a histone acetyltransferase [11], was clarified by a study in animals by Ralph and coworkers. These authors demonstrated, through the lesion of the SCN and the subsequent transplantation, the role of CLOCK in triggering from its hypothalamic site, the rhythms and synchronizing the action of satellite genes in peripheral tissues to harmonize circadian periodicity [13]. Subsequent studies discovered in Drosophila Melanogaster the Period gene (PER) and the PER activator in the mouse. A protein encoded by PER was demonstrated to be able to repress its own transcription, thus promoting PER rhythm [2,13,14]. 

Studies in mouse contributed to clarifying the chain of events leading to the circadian rhythmicity and the related genes involved. This chain is triggered by the main pace-maker CLOCK (circadian locomotor output cycles kaput) and includes, in a negative feed-back loop, some activators that are able to induce the expression of their own repressors [2,15]. This loop includes (BMAL1 (brain and muscle Arnt-like protein1), CRY (cryptochrome), PER (period), RORs (retinoic acid-related orphan receptors), and REV-ERBs (members of the nuclear receptor superfamily of transcription factors) [2,16,17,18,19]. In particular, CRY plays an important role in this network, especially in metabolic processes, mediating the circadian regulation of cAMP signaling and hepatic gluconeogenesis [20]. In fact, CRY, interacting with the small molecule KL001, prevents its ubiquitin-dependent degradation, thus allowing its stabilization, which is able to inhibit gluconeogenesis in primary hepatocytes, to lower blood glucose concentrations and to improve insulin sensitivity. The results of these studies pave the way for a possible therapeutic benefit of compounds that enhance CRY activity in type 2 diabetes [20,21]. Recent studies have demonstrated that CRY1 expression is androgen-responsive and is associated with a poor outcome in prostate cancer. The mapping of the CRY1 cistrome and transcriptome revealed that CRY1 regulates DNA repair. The results of these studies identified CRY1 as a pro-tumorigenic factor and as a new possible target in cancer treatment [22].

### 2.3. Synchronization Schedule and Chronotype

Endogenous and exogenous factors may influence the characteristics of circadian rhythms. These factors are classified as “zeitgebers”, entraining agents or synchronizers (Table 1) [6,7,23].

Among the exogenous cues, daily schedules of light/darkness, sleep/wake, periodic food intake and exercise timing can play an important entraining action [23,24,25,26,27,28]. In particular, the light/dark cycle plays an important synchronizing role in endocrine rhythms both directly, by exciting the light-entrainable circadian pacemaker located in the SCN of the hypothalamus, and indirectly, through the variations in melatonin, whose secretion at the pineal level is stimulated by darkness and inhibited by light [29]. The pineal gland plays an intermediate role between environmental stimuli and the endocrine system. Light-feeding phase relations have been shown to play a synergistic role in entraining circadian rhythms in peripheral oscillators [24]. Their phase misalignment may play a desynchronizing action, affecting the central and peripheral clock genes, respectively, causing alterations of hormonal rhythms and metabolic disorders [23,24]. The interconnection between environmental and genetic factors affects the individual chronotype, which is characterized by an individual’s attitude, determining circadian preferences for times of different human activities. An appropriate synchronization is important for ensuring the normal function of physiological processes [30,31]. Even if the chronotypes range from an extremely early (larks) to extremely late (owls) forms, they are usually classified in three general categories: morning, evening, and intermediate chronotypes [32,33,34,35]. The evening chronotype has been associated with several health problems such as psychological disorders, gastrointestinal and cardiovascular diseases, and greater mortality than the morning chronotype [34]; it has also been identified as a risk factor for cancer [35]. Moreover, people with type 2 diabetes and evening chronotypes may be more susceptible to inactivity and poorer glycemic control compared with morning chronotypes. Since chronotype is potentially modifiable, social and lifestyle factors influencing it should be evaluated to optimize the responses to diabetes care. Recent studies have attracted attention towards a role of chrono-epigenetics in the origin of human diseases, highlighting also the interconnections between circadian clocks, epigenetics and cancer [36]. Cytosine variations in particular seem to display deterministic temporal rhythms, which may drive ageing and complex diseases. Recent data suggest that epigenetic changes and chromatin transitions occur in cancer cells, in particular that key chromatin remodelers involved in histone modifications play an important role in the development of cancer [3,36,37]. Rhythmic hormonal variations are regulated by central and peripheral clocks; however, they may affect in a mirror-like way the genetic chrono-pathway by acting as endogenous entraining agents of circadian clocks. In fact, the results of recent studies in vitro have demonstrated the potential resetting mechanism at three levels: the hormone, the direct clock gene target and the tissue clock response [38]. An adequate interconnection between all the factors regulating circadian rhythmicity is fundamental for allowing appropriate mammalian physiology and behavior, because the disruption of circadian harmony at any level may cause disorders in several organs, including the cardiovascular system, with severe consequences for the individual health and with sex-related differences in response to desynchronizing injuries [39,40].

## 3. Endocrine Chronophysiology and Chronopathology

The endocrine system comprises a complex network, including the central and autonomous nervous systems, central and peripheral endocrine glands and the immune system. This network is under control of central and peripheral clocks and is entrained by endogenous and environmental factors to ensure a harmonic physiological circadian chrono-organization [41,42,43]. The main hypothalamic clock orchestrates, in cooperation with other local clocks and with environmental synchronizers, especially the light, the rhythmic secretions of the hypothalamus–pituitary–satellite gland axis [42,43,44,45]. 

### 3.1. Chrono-Organization of the Hypothalamic–Pituitary–Adrenal Axis 

The correct chrono-organization of this axis is important for human health, as it plays an important role in controlling stress responses and regulating the immune system and some psycho-physiological events, such as moods and emotions in tense situations. In particular, the hormones of the hypothalamic–pituitary–adrenal axis are the main mediators of bodily responses to stress, including physical and mental components, so all the conditions that disturb the normal synchronization of these rhythms can negatively affect the human health [44,45].

**Figure 1:**
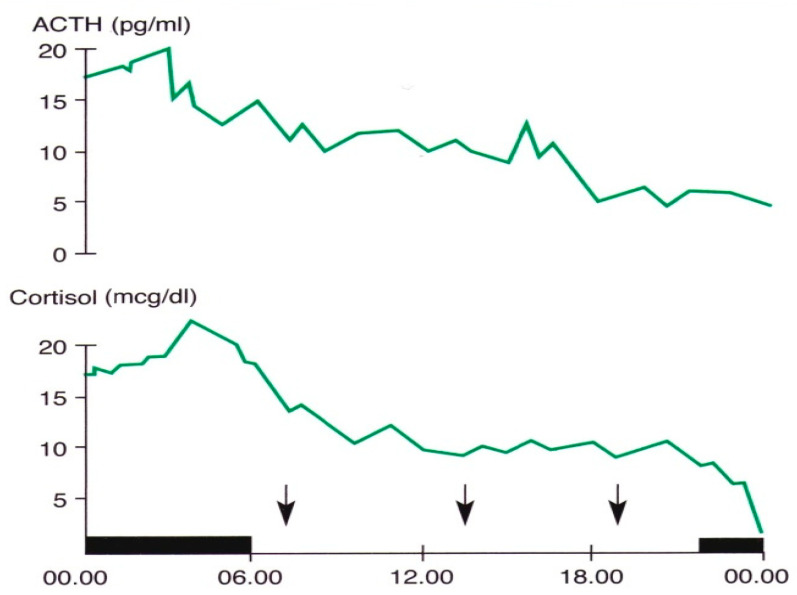
Circadian and ultradian variations in ACTH and cortisol concentrations obtained by subsequent samples in a young man. The black band indicates the sleep period, the arrows the mealtimes (personal observation).

In 1943, Pincus described a diurnal rhythm of urinary ketosteroid excretion in young adults [46]. In subsequent years, several studies were focused on the secretory pattern of the hormones of this axis, and clarified the hierarchical way of the rhythmic secretion of these hormones and the genetic and environmental synchronizing factors of these rhythms [41,42,43,44,45,46,47]. In particular, recent studies in vitro and in vivo have contributed further to the knowledge of this issue. Jones and coworkers demonstrated the role of circadian neurons in the paraventricular nucleus in entraining and sustaining daily rhythms in glucocorticoids [47]. Malek and coworkers, using a mathematical analysis of the role of pituitary-adrenal interactions in ultradian rhythms of the hypothalamic–pituitary–adrenal (HPA) axis, demonstrated the persistence of ultradian adrenocorticotropic hormone (ACTH) and cortisol rhythms in absence of corticotropin-releasing hormone (CRH) stimulation [48]. ACTH and cortisol show circadian and ultradian rhythms with overlapping phases, with the zenith in the morning and the nadir in late evening/night, suggesting a driving ACTH role in these rhythmic variations [6,7,47,48,49] (Figure 1). 

Light is the most important entraining agent of these rhythms: in fact, the lack of light stimulus in totally blind subjects induces an increase in melatonin secretion, and circadian rhythm disorders and complex hormonal alterations in prepubertal, adult and elderly blind subjects [50,51,52,53,54]. Moreover, combined alterations of circadian hormone rhythmicity, including cortisol rhythm, have been shown in people with obesity, even when they have been submitted to intermittent fasting as a losing weight strategy [55,56]. Disruption of circadian rhythms, including cortisol oscillations, usually occurs in shift workers. This results not only in a misalignment of the circadian and external light/dark cycles but may also involve peripheral clock genes and transcripts of other human genomes, with important metabolic alterations [57]. A bidirectional relationship exists between circadian rhythms and mood disorders. Mood disorders are often associated with a disrupted circadian cortisol rhythm, whereas disruption of this rhythm by jet lag, shift work or exposure to night-time artificial light may induce or exacerbate mood disorders in susceptible subjects [58]. Concerning this, very recently, Bilgin et al. investigated the diurnal salivary cortisol in young adults that had had multiple and persistent regulatory problems (sleeping, crying or feeding problems) in their early childhood, and showed an increased HPA axis activity in response to awakening stress and behavior problems in these subjects [59]. A clear daily circadian rhythm has been demonstrated for salivary dehydroepiandrosterone (DHEA), with some sex-related differences and a flatter profile in older age [60], whereas the rhythms of the other adrenal hormones, such as aldosterone (regulated by renin secretion rather than ACTH), adrenaline and noradrenaline, are less characterized. Their circadian variations are strongly influenced in a bidirectional way by metabolic pattern variations and psycho-physical activity, as their action (especially that of adrenaline and noradrenaline) promotes the mobilization of metabolic energy factors that are preparatory to any physical activity. 

### 3.2. Growth Hormone

**Figure 2:**
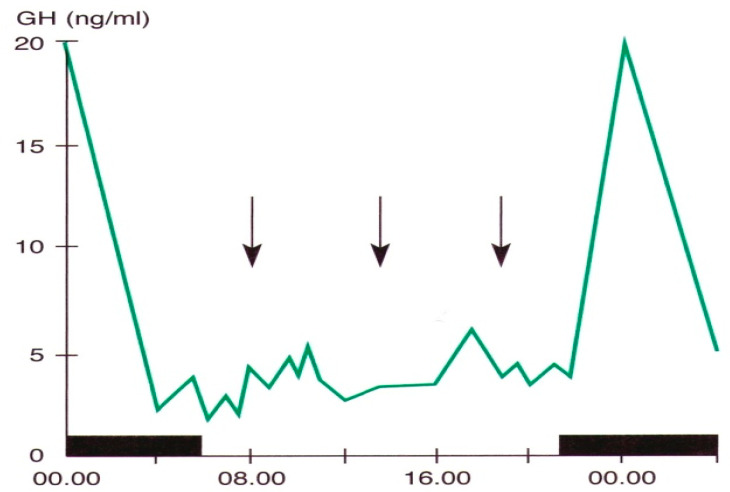
Daily variations in plasma GH levels obtained by samples taken at defined intervals in a young man. The black band indicates the sleep period, the arrows the mealtime (personal observation).

Pituitary growth hormone (GH) secretion is under hypothalamic control through a balance between a stimulatory (Growth Hormone-Releasing Hormone, GHRH) and an inhibitory (somatostatin) factor. Sleep is the most important entraining agent for circadian GH variations; the highest hormone secretory peak is reached during the deep sleep phase [5,7,25,61] (Figure 2). 

This peak usually makes its onset in both sexes from the 3rd month of life and reaches its maximum expression at puberty and in post-pubertal phases. It then persists with the same characteristics during adulthood and decreases to a flattened profile in senescence. The nocturnal GH increase is linked to some electro-encephalographic phases of sleep [25] and this correlation is maintained even during inverted sleep/wake rhythms. Some lower peaks may be observed during inter-meal intervals and during afternoon naps. Even if sleep plays a pivotal role in regulating the daily variations in GH secretion, a complementary synchronizing role of the light/dark cycle on this secretion may not be excluded, considering that in blind subjects the nocturnal peak is lacking, the response of GH to L-Dopa stimulated test is impaired, and prepubertal subjects with total blindness show impairment of body growth [7,61,62,63]. Moreover, disorders of GH secretion with impaired or absent nocturnal peak, have been described in obese patients, in patients with hypothalamic–pituitary diseases, and in prepubertal and adult patients with GH deficiency [5,8]. GH plays several actions other than that on the growth. In particular, it is involved in the regulation of intermediate metabolism, and in the trophism and function of various organs and systems, especially the cardiovascular, muscular and bone systems, throughout life. Therefore, if a secretory deficiency in a developmental age is responsible for short stature, a deficiency in adulthood characterizes a nosographic picture classified as adult GH deficiency syndrome, characterized by cardiac, muscular, bone, metabolic and psychic alterations [64]. An interesting connection between GH variations and the HPA axis has recently been demonstrated. The GH receptor (GHR) is present in CRH cells, which are the dominant neuronal population responsive to GH in the paraventricular nucleus of the hypothalamus. However, studies in mice have demonstrated that GHR ablation in CRH-expressing neurons causes reduced energy expenditure but does not lead to major alterations in metabolism, in the HPA axis, in acute stress response or anxiety [65]. Even if GH action is exerted at peripheral level through mediation by some growth factors, especially insulin-like-growth factor 1 (IGF1), mainly produced by the liver, the daily variations in IGF1 are not as well coded as those of GH.

### 3.3. Prolactin

**Figure 3:**
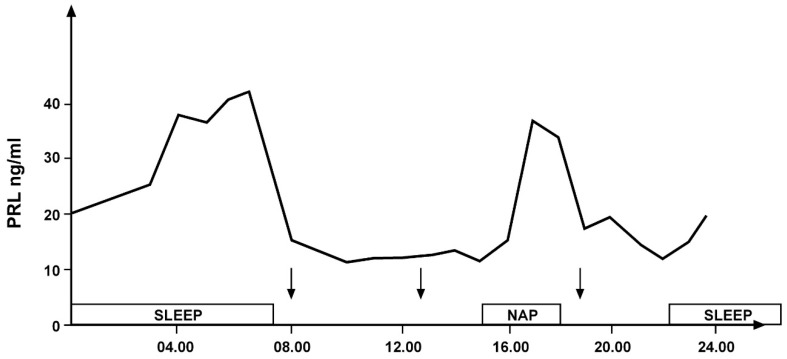
Daily variations in prolactin secretion in a young healthy woman: secretory peaks during nocturnal sleep and an afternoon nap indicated by the empty bands. The arrows indicate the time of meals (personal observation).

Similar to GH secretion, rhythmic secretion of prolactin (PRL) is also closely related to sleep. An increase in PRL levels occurs both during nocturnal sleep and during a daytime nap [7,8] (Figure 3). 

Estrogen variations in women play a further synchronizing role in prolactin secretion, by affecting both circadian and infradian prolactin rhythmic secretions. The amplitude of secretory prolactin peaks is higher in females than in males, reaching the highest frequency and amplitude in correspondence with the increased estrogen levels in the pre-ovulatory period of the circatrigintan menstrual cycle [7,8]. Moreover, a circannual prolactin rhythm with secretory zenith in March/April has been described in prepubertal girls but not boys [66]. PRL is also involved in the complex stress-evoked cascade of events, with an increase in its levels induced by any stress condition [7,8] and these stress-related variations may negatively affect the rhythmicity of other hormonal secretions, mainly those of the pituitary–gonadal axis. Pathological hyperprolactinemia with disruption of its rhythmic secretion is mainly associated with the presence of a prolactinoma or due to pharmacological effects induced by drugs that interact with the dopamine system. The consequent associated disorders of the hormones of pituitary gonadal axis frequently induce reproductive dysfunction and may lead to infertility in both males and females [67]. 

### 3.4. Hypothalamic–Pituitary–Thyroid Axis

Thyroid hormone (thyroxine, T4, and triiodothyronine, T3) secretion is under control of the hypothalamic–pituitary axis. Hypothalamic thyrotropin-releasing hormone (TRH) stimulates at the pituitary level the release of thyrotropin (TSH), which in turn stimulates the thyroid gland to produce T4 and T3. Thyroid hormones regulate, by negative feedback, the secretion of both TRH and TSH, acting at hypothalamic and pituitary levels, respectively. A further direct inhibiting action on TSH secretion is exerted by somatostatin, through binding to some of its receptor subtypes that are expressed in the pituitary TSH-secreting cells [68,69].

TSH shows circadian and ultradian variations with a secretory zenith in late evening and during the first hours of the night, which usually doubles or quadruples the morning value [7,8,9]. A direct connection with the biological clock in the SCN allows the physiological diurnal oscillations of TSH and of thyroid hormones. Studies in animals suggest a dual control mechanism for thyroid function, involving both TRH-TSH release and thyroid gland secretion [70]. Disorders of daily TSH oscillations have been described in shift workers, elderly subjects, people with obesity and persons with Cushing’s syndrome [71,72]. In subjects with type 1 diabetes mellitus, the daily oscillation of TSH may be inversely correlated with glycemic variations, regardless of the variations in thyroid hormone concentrations [73]. 

Among the environmental entraining agents, the light/darkness cycle also influences TRH-TSH-thyroid hormone secretions both in animals [74,75,76,77,78,79,80] and in man [81,82,83] with a stimulating action by light and an inhibiting action by darkness. TSH, in turn, has been shown to play a pivotal role in the transduction of photoperiodic signal [74]. Studies in mice demonstrated that under short photoperiod, melatonin inhibits the pars tuberalis production of TSHβ, which in turn acts on tanycytes to regulate seasonal control of the intra-hypothalamic thyroid hormone T3. This hormone, through binding to its receptor TRα regulates RFRP-3 neurons thus contributing at synchronizing reproduction with the seasons [75]. The lack of light stimulus in blind subjects also impairs pituitary-thyroid function as well as the secretions of other pituitary hormones and, consequently, the hormones of the related satellite glands [9,29,50,51,52,53,55,81,82]. 

The occurrence of infradian circannual variations in TSH secretion has been validated both in children and in adults with zenith of secretion in winter [83,84,85,86]. These variations seem to be inversely entrained by variations in environmental temperature rather than by variations in thyroid hormone secretions [86]. Furthermore, seasonal secretory variations in thyroid hormone secretions also appear to be inversely related to variations in environmental temperature, as they increase in winter and decrease in summer [87]. An interconnection between infradian TSH variations and mood has been demonstrated in subjects living in Antarctic, with zeniths in November and July and nadirs in March and April. A negative feed-back between mood and FT3 but not FT4 variations was suggested by the decline in only FT3 concentrations following the peak of tension-anxiety [88].

The integrity of circadian chrono-organization is essential for the correct functioning of the immune system [9,89,90]. Therefore, impairment of circadian organization may increase the occurrence of immune diseases, including those of thyroid gland [9,82]. Conversely, thyroid disorders may impair circadian clock. Concerning this, hyperthyroidism increases and hypothyroidism disrupt, the expression of some genes with consequent alterations in TSH but also in other pituitary hormone rhythmicity [91].

Circadian disruption and disorders of clock gene expression may favor the occurrence of several types of cancers, including thyroid cancer [2,3,35,82,92,93,94]. Normal thyroid tissue obtained by biopsy has been shown to express different levels of some clock genes with respect to tissue from thyroid nodules. Different expression in particular of BMAL1 and CRY2 has been found in normal versus malignant thyroid cells [95]. 

Patients with disruption in TSH and T3 rhythmicity related to shift work, jet lag, and chronic sleep disorders show a high prevalence of malignancy, including thyroid cancer [82]. Considering this, it is mandatory make every effort to prevent disruptions of the chrono-organization of HPT axis secretions, and to promptly correct them, when occurred, with appropriate measures to avoid the effects of environmental and individual desynchronizing factors thus preventing the possible occurrence of thyroid cancer [96].

### 3.5. Hypothalamic–Pituitary–Gonadal Axis

**Figure 4:**
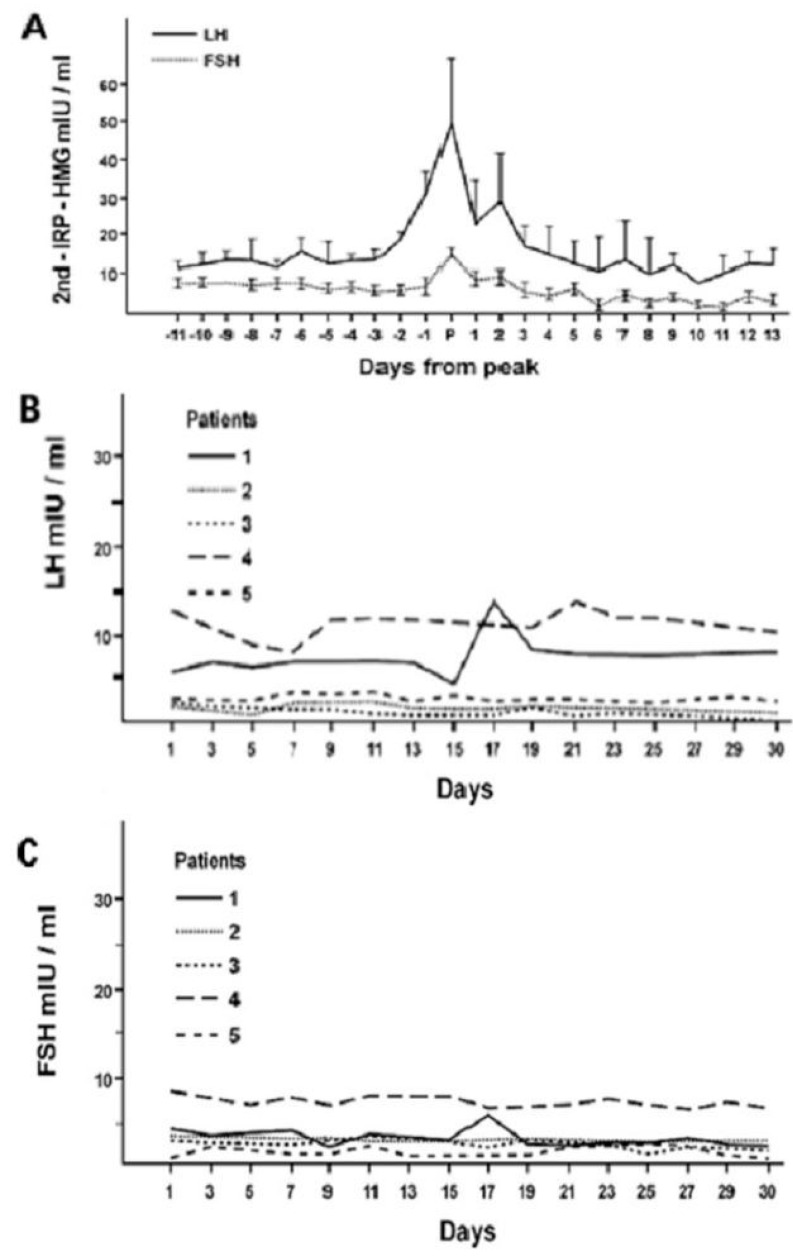
Infradian circatrigintan rhythms of plasma LH and FSH concentrations in healthy women (**A**) and in five women with hypogonadotropic amenorrhea (**B**,**C**): occurrence of the ovulatory gonadotropin peak in women with normal menstrual cycle (**A**) and its absence from patients with amenorrhea (**B**,**C**) (personal observation).

The secretory patterns of the hormones of this axis exhibit ultradian and infradian, and, less characterized, circadian rhythmicity, which is conditioned by phenotypic sex and regulates the stages of development, sexual maturation and senescence. The onset of puberty is precisely announced by the appearance of the pulsatile activity (ultradian rhythm) of the gonadotropins, especially LH, an activity that becomes increasingly marked with the progression of the pubertal stages [7]. In young women, after the completion of pubertal development, the classic infradian menstrual rhythm appears with the characteristic ovulatory peak of LH and FSH, that is lacking in women with hypogonadotropic amenorrhea [1,7,8] (Figure 4). 

Seasonal variations in these hormones are particularly important for sexuality and fertility, also in humans [97]. A circannual rhythm of pituitary and gonadal hormones, already present in the prepubertal age, is consolidated in the adults and presents a secretory acrophase of LH in January (Wintertime in North Hemisphere) in both sexes and of testosterone in late summer/autumn in men [66,97,98]. The occurrence in adult men of circannual LH and testosterone rhythms without overlapping phases but in antiphase (shift of approximately six months = 180°), seems to indicate that the driving role of these rhythms is played by the testis and that testosterone variations regulate by negative feed-back the pituitary infradian LH rhythm [66,99]. This seems to be confirmed by the persistence of a circannual testosterone rhythm in people with hypogonadotropic hypogonadism, and with Klinefelter’s syndrome, even if at lower secretory levels and with a phase shift [97,98,99]. The independence of serum testosterone seasonality from infradian LH fluctuations has been confirmed by a more recent study and attributed by the authors to the changes in environmental temperature and daylight duration [100]. Considering the important physiological role of rhythmic gonadal secretions, daily activities, if possible, should be planned in accordance with the most important synchronizing factors of these rhythms, especially light/dark and sleep/awake cycles. To prevent the disruption of these rhythms is mandatory [100,101,102] as it may cause hypogonadism and infertility in both sexes [97,103,104,105].

### 3.6. Insulin, Leptin, and Ghrelin 

The appropriate chrono-organization of mammalian genes involved in the circadian network and the interaction with daily light/dark cycle and other environmental synchronizing factors are necessary to allow the completion of metabolic processes [106,107,108]. These hormones play a pivotal role in regulating metabolic adaptation to the variations in some environmental factors. 

*Insulin*. Circadian insulin oscillations are strictly correlated with food intake, and contribute, together with leptin and ghrelin, to ensure the best metabolic conditions during daily activities. There is a bidirectional connection between circadian clocks and circadian variations in insulin secretion. On the one hand, the circadian clock dictates the time of the regulation of metabolism [3,108]; and, on the other, the circadian rhythm of circulating insulin concentrations in turn plays an important synchronizing action of some clock genes, in particular *PER1* and *PER2* [107,108,109,110]. In this regard, it has been demonstrated that the loss of circadian insulin oscillations induced by a high-fat diet intake causes the disruption of the rhythmic expression of circadian clock genes in the liver [111]. The relationship between molecular oscillators and the secretory pattern of insulin, proinsulin and glucagon, has been recently clarified by Petrenko and coworkers through a study on intact islet cells and islet cell obtained from donors with T2D. Their results demonstrated a reduced insulin and glucagon exocytosis induced by dampening of circadian oscillators [11]. Disorders of circadian machinery, involving in particular BMAL1, may trigger an endocrine adaptation involving GH and sex hormone pathways, leading to insulin resistance and liver disease but also, in some cases, hypo-insulinemia and diabetes [112,113,114,115,116].

*Leptin*. This hormone is the product of the ob gene and is secreted by adipocytes. It is involved, in co-operation with ghrelin, insulin and orexin, in the regulation of the daily rhythmicity of blood glucose and food intake, body weight, energy homeostasis and a wide spectrum of biological activities [116]. Leptin has also been shown to play a role in expression of some genes; it causes up-regulation of *PER2* and *CLOCK* gene expression in mouse osteoblasts, which have an endogenous circadian clock [117]. Leptin exhibits a circadian rhythm in humans with the highest concentrations during the night and the lowest during the day, strictly correlated with metabolic processes [118,119]. A significant circadian variation in leptin concentrations is found whatever the age, with a peak at night and through around noon, but at higher secretory levels in older subjects with normal-weight and with overweight [120]. Among the extra metabolic activities of leptin, of particular importance is the chrono-regulation of the hypothalamus–pituitary axis. A normal rhythmic secretion of leptin is necessary for triggering pulsatile gonadotropin secretion, essential for pubertal development. However, an excessive level of leptin may negatively affect the hypothalamic–pituitary–gonadal axis function, as demonstrated in subjects with increased body mass index [121]. Moreover, a disruption of circadian rhythm of leptin concentrations has been described in people with critically ill heart failure [122].

*Ghrelin*. The pivotal role of this hormone in regulating metabolic processes was ascertained sometime after its identification in 1999 as a GH-releasing peptide. Plasma ghrelin concentrations in humans show circadian variations strictly correlated with meal times, reaching the highest levels before and the lowest after food ingestion, and showing stable high levels during sleep. This suggests a role of this hormone in meal initiation, with stimulation of appetite through the activation of orexigenic hypothalamic neurocircuits [123]. The concomitant food-intake-independent stimulation of lipogenesis, associated with the ghrelin action on glucose metabolism [124], favors an increase in body weight and obesity. A recent study on the circadian variations in ghrelin, leptin and appetite in lean adults kept under controlled diurnal conditions of synchronization with respect of day/night, sleep/wake and light/darkness cycles, showed that all these variables exhibited circadian variations. In particular, unacylated ghrelin showed a rhythm with acrophase occurring shortly after waking and a nadir in evening, whereas leptin showed a rhythm with acrophase occurring shortly after lights-out and nadir at midday [125]. Finally, ghrelin, in association with melatonin, plays a synchronizing role on the sleep–wake cycle, which favors adequate physical activity, establishing a virtual circle with physical exercise [126].

## 4. Elementary Principles of Chronotherapy

The fundamental principles on which chronotherapy is based are the following: cts.

- Maintain an optimal circadian organization of the individual to be treated;
- Timing the administration of drugs and targeting the biological clock;
- Replacement therapy carried out, if possible, mimicking the circadian rhythm of the variable to be replaced;
- Looking for and use of chronobiotic drugs capable of recovering desynchronized rhythms.

Several studies have demonstrated that the intensity of therapeutic effects and the severity of side effects of some drugs vary, depending on the time they are administered [127,128,129,130]. In particular, a chronotherapeutic approach in treatment of malignancy has been shown to improve the host tolerance when it is performed according to host rhythms [127]. The respect of the chronotherapy canons is very important in subjects with hormonal deficiencies from endocrine diseases, to whom the hormone replacement therapy has to be aimed not only at bringing the values back to the normal range but also, if possible, at restoring the lost circadian, ultradian and infradian rhythm of the hormone to be replaced. Corticosteroid replacement therapy in individuals with adrenal insufficiency is a classic example of circadian chronotherapy, because it is usually carried out by administering the highest dose in the morning and the lowest in the afternoon/evening or by administering a once-daily, modified-release hydrocortisone that provides a time-related release, mimicking the circadian rhythm of the hormone [7,8,131,132]. In particular, the once-daily modified-release regimen has been shown to reduce body weight and recurrent infections and normalize the immune cell profile by entraining clock genes in immune cells, thus improving the quality of life of treated patients [131,132]. Following the canons of chronotherapy, GH replacement treatment in subjects with GH deficiency should also involve administering the hormone in late evening to favor the plasma nocturnal increase that mimics the normal circadian pattern of this hormone. Moreover, considering the physiological role of infradian testosterone variations in men, testosterone replacement therapy in male with hypogonadism, in addition to ensuring the recovery of plasma testosterone concentrations to the normal range, should be aimed also at restoring, if possible, the circannual rhythm of this hormone, which is altered in these patients [98,99,100].

Considering the harmful effects of rhythmic disruption, it is essential to look for chemical or natural products that can exert a chronobiotic action in regulating internal biological clocks. These products have to be able to restore a previously disrupted rhythm that still persists after correcting environmental desynchronizing factors. Among the hormones playing a chronobiotic role, melatonin has certainly to be considered: it is able to reset the sleep-wake rhythm when administered in the late evening, and to prevent the jet lag syndrome when administered before transoceanic travel [133,134]. Moreover, it has been shown, in relationship with leptin and adiponectin, to favor the resynchronization of rhythm disruption in obesity [135] and to restore the circadian misalignment caused by optic neuritis [136].

Ghrelin has also been shown to have a chronobiotic action. Cultured hepatocytes from steatotic liver treated with ghrelin show a recovery of their circadian pattern of clock genes such as BMAL1, CLOCK and PER, previously blunted by steatosis [96].

Promising results have been obtained by the use of nobiletin as a natural chronobiotic factor. This polymethylated flavone with the greatest abundance in citrus peels is able in enhancing circadian rhythms [137,138], directly targeting the molecular oscillator, and, by this action, protecting against metabolic syndrome [137]. The chronobiotic action of nobiletin was shown: (i) to promote bioenergetics and healthy aging [139], (ii) to mitigate astrogliosis-associated neuroinflammation and disease hallmarks in an Alzheimer’s disease model [140], and (iii) to play a multifunctional role in cancer chemoprevention [141]. More recently, a chronobiotic role has been demonstrated for another polymethoxyflavone, sudachitin, which has been shown to modulate the circadian CLOCK and improve liver physiology [142].

## 5. Conclusions

Molecular studies have clarified the complex chain of events driven by the master clock located in the suprachiasmatic nucleus that coordinates peripheral clocks and daily expression of their clock and clock-controlled genes. However, a functional chrono-organization is the result of an interplay between the molecular clocks and several endogenous and environmental factors. An important role in physiological processes is played by endocrine rhythms. Disruption of circadian machinery involving hormone secretions may promote several diseases including cancer. Considering the appropriate timing of hormonal circadian rhythmicity, in clinical practice has to be taken into account to avoid desynchronizing conditions that may negatively affect human health. Thus, it is necessary to modulate the therapeutic procedures along the lines of a corrective chronotherapy, considering an appropriate time of drug or hormone administration, to avoid disruption of circadian rhythms, to reduce the dosage, and to eliminate or at least reduce harmful side effects. Finally, further efforts have to be aimed at searching for new chronobiotic agents, in addition to those already known, capable of resynchronizing disrupted rhythms.

## Figures and Tables

**Table 1:** Endogenous and environmental factors that may influence the characteristics of circadian rhythms (Ref. [9], modified).

Light/darkness cycle
Sleep/wake alternations
Periodic food intake
Social environment (physical and mental work, travel)
Family and individual chronotype
Epigenome
Hormone variations
